# The Dominant Role of Recrystallization and Grain Growth Behaviors in the Simulated Welding Heat-Affected Zone of High-Mn Steel

**DOI:** 10.3390/ma17102218

**Published:** 2024-05-08

**Authors:** Yangwen Wang, Honghong Wang, Siyuan Peng, Bin Xia, Hai Zhu

**Affiliations:** The State Key Laboratory of Refractories and Metallurgy, Wuhan University of Science and Technology, Wuhan 430081, China

**Keywords:** high manganese austenitic steel, welding heat-affected zone, recrystallization, grain size, mechanical property

## Abstract

Single-pass-welding thermal cycles with different peak temperatures (T_p_) were reproduced by a Gleeble 3800 to simulate the heat-affected zone (HAZ) of a Fe-24Mn-4Cr-0.4C-0.3Cu (wt.%) high manganese austenitic steel. Then, the effect of T_p_ on the microstructure and mechanical properties of the HAZ were investigated. The results indicate that recrystallization and grain growth play dominant roles. Based on this, the HAZ is proposed to categorize into three zones: the recrystallization heat-affected zone (RHAZ) with a T_p_ of 700~900 °C, the transition heat-affected zone (THAZ) with a T_p_ of 900~1000 °C, and the coarse grain heat-affected zone (CGHAZ) with a T_p_ of 1000~1300 °C. The recrystallization fraction was 29~44% in the RHAZ, rapidly increased to 87% in the THAZ, and exceeded 95% in the CGHAZ. The average grain size was 17~19 μm in the RHAZ, slightly increased to 22 μm in the THAZ, and ultimately increased to 37 μm in the CGHAZ. The yield strength in the RHAZ and THAZ was consistent with the change in recrystallization fraction, while in the CGHAZ, it satisfied the Hall–Petch relationship with grain size. In addition, compared with the base material, the Charpy impact absorbed energy at −196 °C decreased by 22% in the RHAZ, but slightly increased in the CGHAZ. This indicates that the theory of fine grain strengthening and toughening is not entirely applicable to the HAZ of the investigated high-Mn steel.

## 1. Introduction

Recently, the global demand for clean energy such as liquefied natural gas (LNG) has continued to increase [1]. Due to the outstanding cryogenic mechanical properties comparable to 9%Ni steel [2,3] as well as the low cost [4], high manganese austenitic steel with a content of 22~25 wt.% manganese has emerged as a highly promising material for cryogenic applications [5]. At present, previous literature has mainly focuses on the optimization of the chemical composition, microstructure, and heat treatment process of high manganese austenitic steel, with less research on weldability [6]. Therefore, the relatively slow research on weldability hinders the promotion and application of this novel cryogenic steel.

High manganese austenitic steel for cryogenic application has a fully austenitic structure, and the austenite phase can remain stable during welding thermal cycling [7,8]. This leads to significant differences in the HAZ characteristics of high manganese austenitic steel compared with other steels and alloys such as high-strength low-alloy (HSLA) steels [9], austenitic stainless steels [10], high manganese low-density steels [11], nickel-based superalloys [12,13] and high-entropy alloys [14]. Due to the absence of γ ⟶ α phase transformation, the HAZ characteristic of high manganese austenitic steel is significantly different from that of HSLA steel [15]. For example, it is common for an inter-critical heat affected zone (ICHAZ) to exist in the HAZ of HSLA steel, but cannot be found in high manganese austenitic steel. Similarly, γ ⟶ δ phase transformation has been reported to lead to a deterioration in the mechanical properties of the HAZ in austenitic stainless steel [10] and high manganese low-density steel [11], however, it is difficult to find in high manganese austenitic steel. A common factor that deteriorates the HAZ of high manganese austenitic steel [16,17,18] and austenitic stainless steel [19] is the precipitation of a M_23_C_6_-type carbide caused by the macrosegregation and/or grain boundary segregation of C, Mn, and Cr elements. Meanwhile, in regions depleted in solutes for high manganese austenitic steel, the ε/α′ phase may occur due to low stacking fault energy [20]. On the one hand, the hard and brittle ε/α′ phase was believed to damage the impact toughness of high manganese austenitic steel [21]. On the other hand, the transformation-induced plasticity (TRIP) mechanism related to strain-induced γ ⟶ ε phase transformation was used to regulate mechanical behavior in high-entropy alloys [14]. High temperature alloys are strengthened by the γʹ phase [13], while high manganese austenitic steels generally have a fully austenitic structure, so strengthening phases are not considered in the HAZ of high manganese austenitic steels. Therefore, our previous understanding and experience of the HAZ in other steels and alloys cannot be directly applied to the study of weldability in high manganese cryogenic steel.

In addition, there are common concerns regarding the welding HAZ of high manganese austenitic steel for cryogenic application. The variation in grain size leads to the inhomogeneous mechanical properties of the HAZ [22,23], and unrecrystallized grains result in performance degradation due to residual stress [24,25]. However, although these studies mentioned the factors that affect the microstructures and mechanical properties of the HAZ of high manganese steels, there is a lack of systematic and qualitative research on the characteristics of the HAZ. This is obviously not conducive to the promotion and application of this novel cryogenic steel.

In this study, single-pass-welding thermal cycles with different peak temperatures (T_p_) were reproduced by a Gleeble 3800 to simulate the HAZ of a hot-rolled high manganese austenitic steel with a partially recrystallized austenite structure. Our purpose was to reveal the crucial factors affecting the evolution of the microstructure and mechanical properties of the HAZ and to discuss the inherent correlation between the two. Based on this, the specialized classification of the HAZ can be realized and is of great significance for further research on the weldability of high manganese austenitic steel in the future. Meanwhile, this fundamental research provides a reference for the formulation of welding process parameters.

## 2. Materials and Methods

### 2.1. Materials

The high manganese austenitic steel (hereinafter to be referred as high-Mn steel) used in this study was a 21 mm thick hot-rolled steel plate manufactured by Nanjing Iron and Steel Co. Ltd. (Nanjing, China). Its nominal composition was approximately 0.4C, 24Mn, 4Cr, and 0.3Cu by weight percentage, with the rest being Fe.

### 2.2. Welding Thermal Cycle Simulation

**Figure 1:**
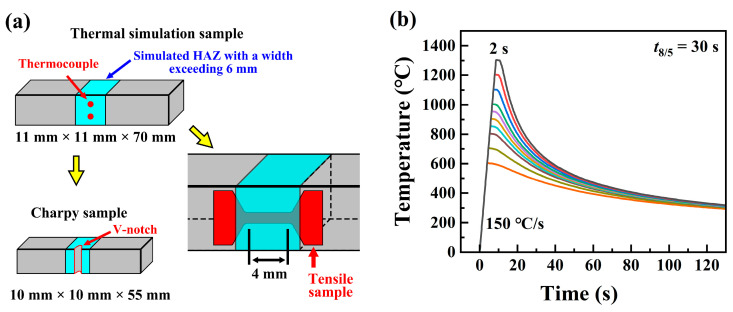
(**a**) Processing schematic diagram of the thermal simulation samples. (**b**) Temperature–time curves during the welding thermal cycle.

The Gleeble 3800 (DSI, America) was utilized to prepare the simulated samples by welding thermal cycles. The dimensions of the samples were 11 mm × 11 mm × 70 mm, with the length aligned parallel to the rolling direction. The thermocouple was controlled in the center of the thermal simulation samples. After the thermal simulation, the samples were further processed into Charpy impact samples and tensile samples, as shown in Figure 1a. The simulated peak temperatures (T_p_) of the welding thermal cycle were 600 °C, 700 °C, 800 °C, 850 °C, 900 °C, 950 °C, 1000 °C, 1100 °C, 1200 °C, and 1300 °C, respectively. Lee et al. [26] determined the maximum formation temperature of a M_23_C_6_-type carbide to be within the range of 600~890 °C through thermodynamic calculations for Fe-0.45C-24Mn-(0, 3, 6)Cr (wt.%) steel with a similar chemical composition. Meanwhile, they reported that no carbide was observed after aging the steel at 450 °C for 1 h. Therefore, the minimum T_p_ of 600 °C was chosen for this preliminary research. The maximum T_p_ was set to 1300 °C, which was below the melting point of approximately 1400 °C for the high-Mn steel. A constant heating rate of 150 °C∙s^−1^ was applied, with the dwell time of 2 s at T_p_. The cooling time from 800 °C to 500 °C (*t*_8/5_) was set to 30 s, and the corresponding heat input was calculated as 24.6 kJ/cm, according to the process outlined in Ref. [27]. The visual representation of the thermal cycling processes is provided, as shown in Figure 1b.

### 2.3. Mechanical Properties Measurement

The hardness was measured using the Innovatest Falcon 500 (INNOVATEST, the Netherlands) Vickers microhardness tester with a load of 1000 g applied for a duration of 15 s. Each sample was tested ten times to display the average hardness level. Charpy impact tests at −196 °C were conducted on an MTS E22.452 (MTS, America) impact tester, in accordance with the ASTM E23-18 standard [28]. The simulated samples were processed into V-notch Charpy samples with a standard size of 10 mm × 10 mm × 55 mm. Tensile tests were conducted at room temperature using an INSTRON 5587 machine (INSTRON, Britain) with a constant crosshead speed of 0.5 mm∙min^−1^ (~2 × 10^−3^ s^−1^). Tensile samples were extracted from the simulated samples with a thickness of 1.3 mm and a gauge length of 4 mm. The tensile direction was aligned parallel to the rolling direction. Entire tensile and Charpy tests were conducted three times for each datum.

### 2.4. Microstructure Characterization

The X-ray diffraction (XRD) measurement using XRD-7000 (SHIMADZU, Japan) with a Cu target was conducted to detect the phase composition of the high-Mn steel at room temperature. The step size was set as 0.02°. The cyclic differential scanning calorimeter (DSC) examination with an STA 449 F5 (NETZSCH, Germany) using argon protective media was performed to investigate the solid-state phase transformation of the high-Mn steel during heating and cooling. The testing temperature ranged from 20 °C to 1200 °C and the scanning rate was set to 20 °C∙min^−1^. The microstructure of the simulated samples was observed using a Nova 400 Nano SEM scanning electron microscope (SEM, FEI, America) and Hikari XP electron backscatter diffraction (EBSD, EDAX, America). The JOEL JEM-2100 transmission electron microscopy (TEM) was performed on the sample simulated at the peak temperature of 800 °C. The operate voltage was set to 200 kV.

## 3. Results

### 3.1. Microstructure of the Investigated High-Mn Steel

**Figure 2:**
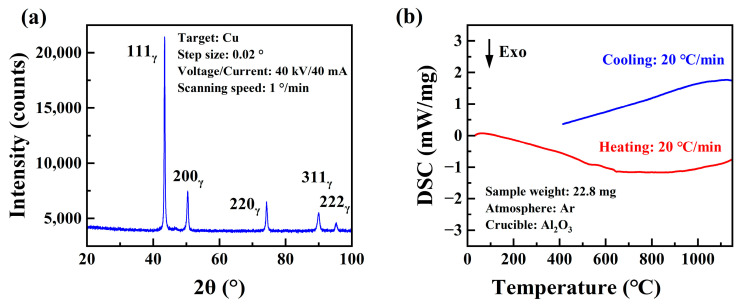
(**a**) X-ray diffraction pattern and (**b**) DSC curves of the Fe-24Mn-4Cr-0.4C-0.3Cu (wt.%) steel.

The XRD pattern obtained from the investigated high-Mn steel sample before the tensile test is visualized in Figure 2a and revealed the presence of only the austenite phase. Figure 2b illustrates the phase transformation characteristics during heating and cooling in the temperature range of 20~1100 °C through the DSC experiment. No significant exothermic or endothermic peaks were observed in the DSC curves. This suggests that there was no significant γ ⟶ ε phase transformation or carbide precipitation in the investigated high-Mn steel during both the heating and cooling processes. Through an appropriate composition design, high manganese austenitic steel can maintain a stable austenite phase at high temperatures [29].

**Figure 3:**
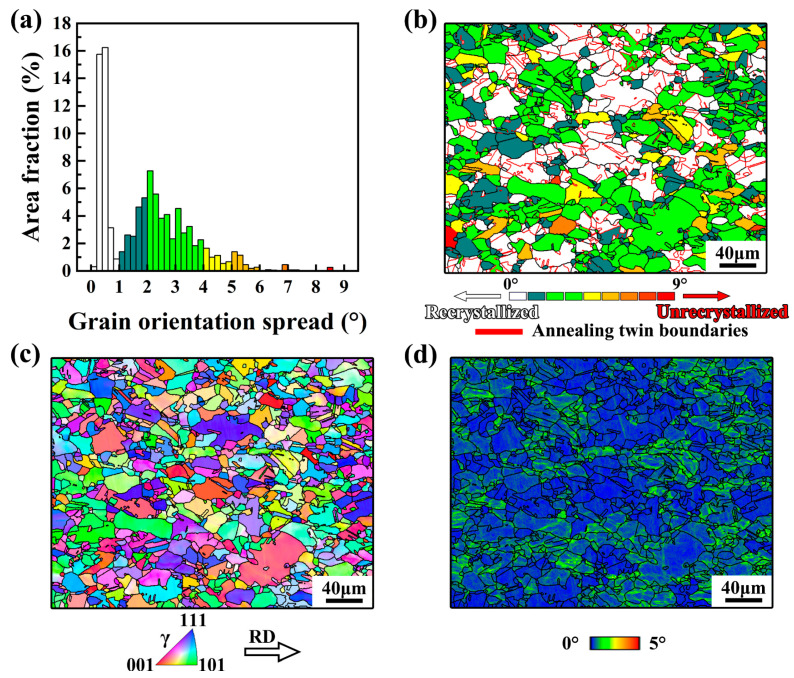
(**a**) EBSD grain orientation spread (GOS) histogram and (**b**) corresponding GOS map, (**c**) inverse pole figure (IPF), and (**d**) kernel average misorientation (KAM) map of the Fe-24Mn-4Cr-0.4C-0.3Cu (wt.%) steel.

The investigated high-Mn steel exhibited a partially recrystallized austenite structure. Figure 3 shows the EBSD analysis. The grain orientation spread (GOS) indicates the degree of grain recrystallization by comparing the local orientation at each analyzed point with the orientation of the neighboring points [25,30]. A low GOS value represents a high degree of recrystallization. The GOS histogram in Figure 3a revealed a bimodal distribution of GOS values with a canyon position at 1°. Grains with GOS values below 1° were categorized as fully recrystallized grains, while grains with GOS values exceeding 1° were classified as unrecrystallized grains. The GOS map in Figure 3b provides insight into the level of recrystallization of the high-Mn steel. The volume fraction of recrystallized austenite grains was measured to be 36.3%. Additionally, the fraction of the annealing twin boundary was measured to be 20.6%. Considering the annealing twin boundaries, the average grain size measured by EBSD was approximately 17 μm.

According to the EBSD inverse pole figure (IPF) demonstrated in Figure 3c, the significant misorientation was observed inside the unrecrystallized grains compared to the recrystallized grains. Furthermore, a kernel average misorientation (KAM) map was employed to illustrate the distribution of strain or dislocations within the unrecrystallized grains [31]. The concentration of strain caused by high-density dislocations was primarily localized near the grain boundaries within the unrecrystallized grains, as depicted in Figure 3d. Similar unrecrystallized grains exhibiting a high density of dislocations were demonstrated to be a “hard phase” with pronounced strengthening effects [2,32].

### 3.2. Mechanical Properties after Thermal Cycling

**Figure 4:**
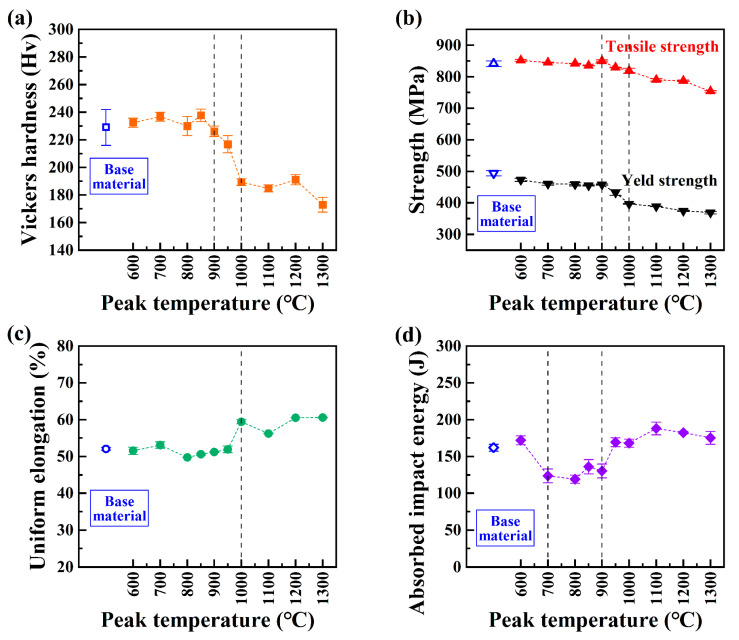
Effect of the peak temperature on (**a**) the microhardness, (**b**) yield strength and ultimate tensile strength, (**c**) the uniform elongation, and (**d**) the absorbed impact energy at −196 °C of the simulated samples by welding thermal cycle.

Figure 4 presents the mechanical properties of the samples simulated at the different peak temperatures (T_p_) of the welding thermal cycles. The average microhardness of the simulated samples exhibited a distinct step-wise variation with T_p_, as illustrated in Figure 4a, and was categorized into the upper shelf, lower shelf, and a narrow transition zone situated between the two shelves. In the upper shelf with a T_p_ of 600~900 °C, the hardness was 226~237 HV, which was similar to the base material’s hardness of ~229 HV. In the lower shelf with T_p_ of 1000~1300 °C, the hardness was 173~191 HV.

Similarly, the effect of T_p_ on the strength of the simulated samples showed a step-wise variation, as illustrated in Figure 4b. Overall, the yield strength and ultimate tensile strength showed a decreasing trend as the T_p_ increased. The upper shelf of strength in the simulated samples showed a slight variation, similar to that of the base material (yield strength of 494 MPa and ultimate tensile strength of 841 MPa). The highest yield strength and ultimate tensile strength were observed at the lowest T_p_ of 600 °C, measured as 473 MPa and 852 MPa, respectively. The lowest yield strength and ultimate tensile strength were recorded at the highest T_p_ of 1300 °C, measured as 369 MPa and 754 MPa, respectively. The strength and hardness rapidly decreased in the T_p_ of 900 and 1000 °C.

The effect of T_p_ on the uniform elongation of the simulated samples was also found to demonstrate a step-wise variation, as illustrated in Figure 4c. The T_p_ range of the lower shelf of uniform elongation was 600~950 °C, slightly wider than that of the upper shelf of strength and hardness. In the lower shelf, the uniform elongation was approximately 51%, which was close to 52% of the base material. Then, the uniform elongation increased to 59% in the upper shelf with a T_p_ of 1000~1300 °C.

The effect of T_p_ on the impact toughness of the simulated samples at −196 °C is displayed in Figure 4d. For the base material, the impact toughness at −196 °C was 162 J. The impact toughness of the sample simulated at the T_p_ of 600 °C was 173 J, which was close to that of the base material, and decreased to 119~136 J in the T_p_ range of 700~900 °C. A decrease of approximately 22% in the minimum absorbed impact energy value was found to be 119 J at the T_p_ of 800 °C. In the high T_p_ range of 950~1300 °C, the absorbed impact energy increased to a level of 168~188 J, which was slightly higher than that of the base material.

### 3.3. Microstructure Evolution after Thermal Cycling

**Figure 5:**
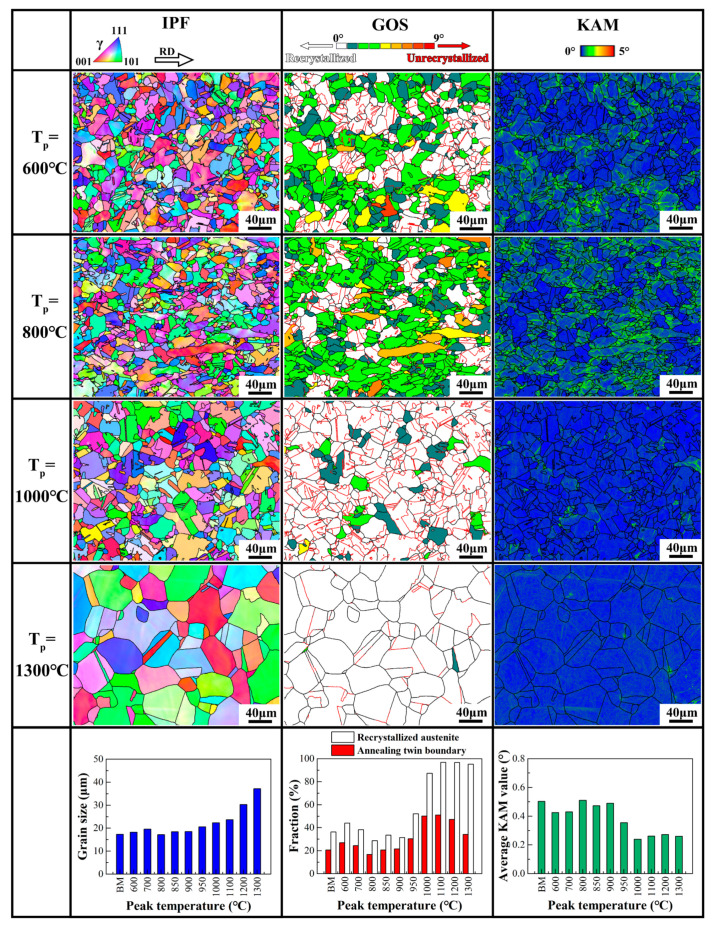
EBSD inverse pole figures (IPF), grain orientation spread (GOS) maps, and kernel average misorientation (KAM) maps of the Fe-24Mn-4Cr-0.4C-0.3Cu (wt.%) steel after thermal cycle simulation at 600 °C, 800 °C, 1000 °C, and 1300 °C. Effect of the peak temperature on the average grain size, recrystallized austenite fraction, annealing twin boundary fraction, and average KAM values of the welding thermal cycle simulation samples are summarized.

The microstructural evolution caused by welding thermal cycles with different peak temperatures (T_p_) was analyzed by EBSD, as illustrated in Figure 5. The average grain size of the simulated samples ranged from 17 μm to 19 μm at the T_p_ range of 600 °C to 900 °C, which was comparable to that of the base material. The recrystallization fraction experienced a rapid increase at the T_p_ between 900 °C and 1000 °C, reaching 87% at the T_p_ of 1000 °C. Meanwhile, the average KAM value decreased to approximately 0.24°, whereas the average grain size exhibited a slight increase from 19 μm to 22 μm. At the T_p_ of 1100 °C to 1300 °C, the recrystallization fraction exceeded 95%, while the average KAM value remained consistently at a low level of 0.24° to 0.27°, indicating the completion of recrystallization. The grains were observed to coarsen as the T_p_ increased, with a maximum grain size of 37 μm at the T_p_ of 1300 °C. Additionally, it was observed that annealing twins developed in the recrystallized grains. Thus, the annealing twin boundary fraction and recrystallization fraction appeared to be synchronized.

**Figure 6:**
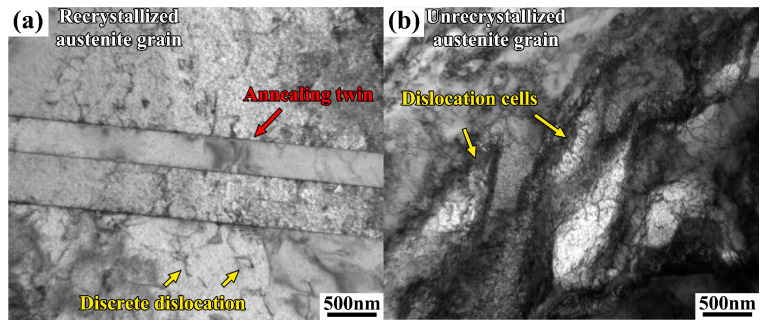
TEM images of (**a**) recrystallized austenite grains with annealing twins and (**b**) unrecrystallized austenite grains with dislocation cells of the Fe-24Mn-4Cr-0.4C-0.3Cu (wt.%) steel after thermal cycle simulation at 800 °C.

TEM images of the recrystallized and unrecrystallized grains in the sample simulated at the T_p_ of 800 °C are presented in Figure 6a,b, respectively. Figure 6a demonstrates that the recrystallized grains, characterized by well-developed annealing twins, contained discrete dislocations distributed within the austenitic matrix. This observation corresponded to the lower KAM values observed in the recrystallized grains (Figure 5). The unrecrystallized grains, on the other hand, exhibited high-density dislocations formed from the dislocation cells, which corresponded to the higher KAM values observed (Figure 5). Furthermore, no carbide was observed in the sample subjected to the welding thermal cycle at the T_p_ of 800 °C. This agrees with the result of the DSC experiment shown in Figure 2b.

## 4. Discussion

### 4.1. Effect of Microstructure by Welding Thermal Cycles on Mechanical Properties

As shown in Figure 2 and Figure 6, no phase transformation took place in the investigated high-Mn steel during welding thermal cycles including martensitic transformation and carbide precipitation. Recrystallization and grain growth play dominant roles. The behavior of recrystallization exhibited relatively low activity below the T_p_ of 900 °C (Figure 4). Then, the recrystallization behavior was pronounced in the T_p_ range of 900~1000 °C and finished above the T_p_ of 1000 °C. After recrystallization was completed, significant grain coarsening occurred. These phenomena account for the observed variations in hardness, strength, and uniform elongation. Hence, in the HAZ of the investigated high-Mn steel, the behaviors of recrystallization and grain growth during the welding thermal cycles played dominant roles in determining the microstructure and mechanical properties.

**Figure 7:**
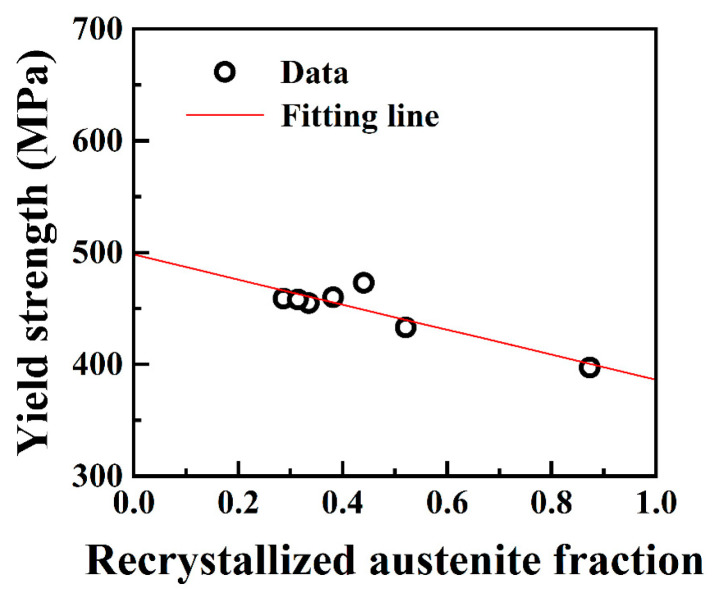
Recrystallized austenite fraction dependence of the yield strength of the Fe-24Mn-4Cr-0.4C-0.3Cu (wt.%) steel after thermal cycle simulation at 600 °C, 700 °C, 800 °C, 850 °C, 900 °C, 950 °C, and 1000 °C.

The partially recrystallized microstructure can be regarded as a mixed structure consisting of recrystallized and unrecrystallized grains. The recrystallized grains are considered as the “soft phase”, while the unrecrystallized grains with high-density dislocations are considered as the “hard phase”. This mixed structure in high-Mn steel provides an effective balance of strength and ductility [2,25,32,33]. Consequently, the hardness and tensile strengths of the simulated samples are proposed to be significantly affected by the recrystallization fraction. The yield strength of the mixed structure follows the rule of mixture [32,34,35,36]:

(1) $$\sigma_{y}=f_{r}\sigma_{r}+\left(1-f_{r}\right)\sigma_{u}$$

where *σ*_y_ is the yield strength of the simulated sample, *f*_r_ is the recrystallization fraction, *σ*_r_ is the yield strength of recrystallized grains, and *σ*_u_ is the yield strength of unrecrystallized grains. Assuming that *σ*_r_ and *σ*_u_ are constants, the relationship between *σ*_y_ and *f*_r_ was fitted based on Equation (1). Figure 7 demonstrates a strong linear relationship between *σ*_y_ and *f*_r_. Additionally, the fitted values for *σ*_r_ and *σ*_u_ were 386 MPa and 498 MPa, correspondingly. Thus, it was confirmed that the recrystallization behavior predominated in the variation in yield strength for the simulated samples at a T_p_ below 1000 °C.

**Figure 8:**
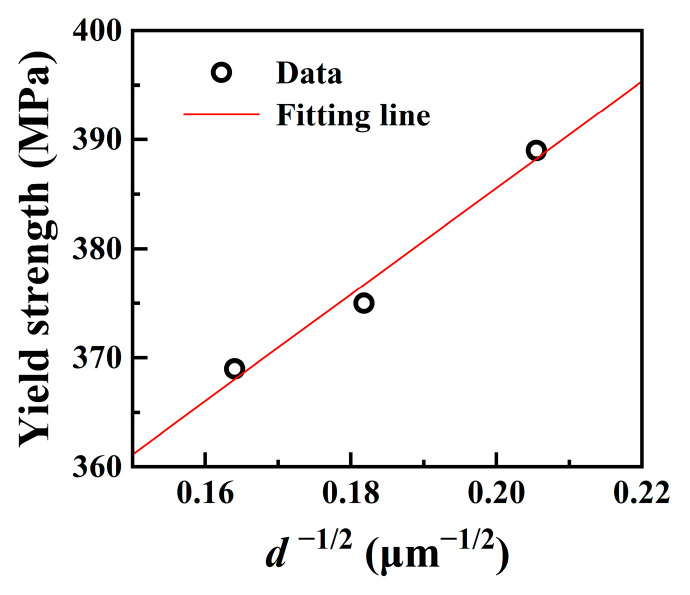
Grain size dependence of the yield strength of the Fe-24Mn-4Cr-0.4C-0.3Cu (wt.%) steel subjected to the welding thermal cycle at the peak temperature of 1100 °C, 1200 °C and 1300 °C.

For the simulated samples at the T_p_ of 1100 °C, 1200 °C, and 1300 °C, the microstructure was fully recrystallized. Here, their yield strength *σ*_y_ is equivalent to *σ*_r_. The variation in *σ*_r_ meets the Hall–Petch relationship [37]:

(2) $$\sigma_{r}=\sigma_{0}+Kd^{-\;\frac{1}{2}}$$

where *d* and K are the average grain diameter and the Hall–Petch coefficient, respectively. *σ*_0_ includes the lattice fraction stress, the solution strengthening contribution of the alloying elements, and the contribution of the initial dislocation density. As shown in Figure 8, there was a strong linear relationship between *σ*_r_ and *d*^−1/2^, disregarding the variations in *σ*_0_. The fitted values for *σ*_0_ and K were determined as 288 MPa and 488 MPa∙μm^1/2^, respectively. Thus, for the simulated samples subjected to the welding thermal cycles at the peak temperature above 1000 °C, the changes in yield strength were primarily influenced by the behavior of grain growth. Additionally, according to Equation (2), the yield strength of the fully recrystallized structure with a grain size of 19 μm was determined as 400 MPa. This value is higher than the yield strength of *σ*_r_ (386 MPa). This deviation is suggested to be attributed to the assumption that *σ*_0_, *σ*_r_, and *σ*_u_ are constants. For instance, the dislocation density in the simulated samples inevitably changes at different T_p_, which results in the changes in *σ*_0_, *σ*_r_, and *σ*_u_.

The changes in hardness with the T_p_ precisely corresponded to the changes in the strength and suggests that hardness testing is expected to be a convenient and reliable method for assessing the strength of the heat-affected zone of high-Mn steel in future applications. The uniform elongation increased from 51% to 59% as the T_p_ exceeded 1000 °C and the recrystallization was completed. Although the grain size continued to increase above 1000 °C, there was almost no change in the uniform elongation. This indicates that grain size had little effect on the uniform elongation of the simulated samples for the investigated high-Mn steel.

The cryogenic impact toughness of the simulation samples at −196 °C exhibited a decrease within the T_p_ range of 700~900 °C, as shown in Figure 4. Up to now, the main factor leading to the reduction in cryogenic toughness in the HAZ of high-Mn steels remains unclear. Some research has suggested that the grain boundary segregation and carbide precipitation [16,18,26,33,38] result in the reduction in cryogenic toughness. However, carbide precipitation was not observed by TEM (Figure 5) in the present work. Due to alloy element segregation behavior [39], segregation is expected to occur during the welding thermal cycle in the investigated high-Mn steel. Further systematic experimental research is needed to detect the effect of the segregation behavior of elements on the cryogenic toughness.

### 4.2. Division of HAZ for the Investigated High-Mn Steel

**Figure 9:**
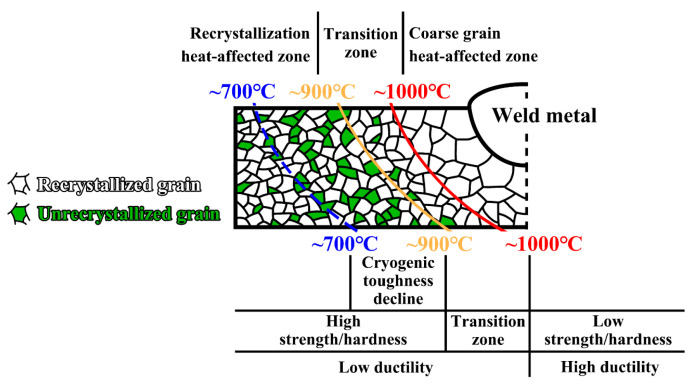
Schematic diagram of the heat-affected zone division based on microstructural and mechanical property evolution.

Based on the aforementioned characteristics of the simulated samples in the investigated high-Mn steel, we proposed that the welding HAZ could be divided into three distinct zones. These zones, as illustrated in Figure 9, include the coarse grain heat-affected zone (CGHAZ), the recrystallization heat-affected zone (RHAZ), and the transitional heat-affected zone (THAZ) between the CGHAZ and RHAZ. The CGHAZ was subjected to a welding thermal cycle with a T_p_ of 1000~1300 °C and was composed of fully recrystallized austenitic coarse grains, characterized by the reduced strength and hardness and good cryogenic toughness. The THAZ experienced the welding thermal cycle with a T_p_ of 900~1000 °C, and the recrystallization fraction of the THAZ underwent a rapid reduction, resulting in a decrease in strength and hardness. The RHAZ was subjected to the welding thermal cycle with a T_p_ of 700~900 °C. The microstructure of the RHAZ was composed of partially recrystallized grains, exhibiting high strength and hardness at room temperature and decreased impact toughness at −196 °C. The ductility remained consistent in both the RHAZ and THAZ, and there was a slight increase in the CGHAZ.

## 5. Conclusions

In this work, the evolution of microstructure and mechanical properties of the current high-Mn steel with peak temperatures (T_p_) was investigated by welding thermal cycle simulation. Some conclusions can be drawn as follows:

(1) No phase transformation was observed in the simulated HAZ. The microstructure and mechanical properties of the simulated HAZ were mainly governed by the recrystallization and grain growth behaviors. Accordingly, we proposed that the HAZ can be classified into three zones: the recrystallization heat-affected zone (RHAZ) with a T_p_ of 700~900 °C, the transition heat-affected zone (THAZ) with a T_p_ of 900~1000 °C, and the coarse grain heat-affected zone (CGHAZ) with a T_p_ of 1000~1300 °C.
(2) The recrystallization fraction was 29~44% in the RHAZ, rapidly increased to 87% in the THAZ, and exceeded 95% in the CGHAZ. The average grain size was 17~19 μm in the RHAZ, slightly increased to 22 μm in the THAZ, and ultimately increased to 37 μm in the CGHAZ. The recrystallization fraction and grain size of the HAZ were primarily influenced by the T_p_ of the welding thermal cycles. The changes in the recrystallization fraction and grain size in the HAZ were closely related to the T_p_ of the welding thermal cycles.
(3) The hardness, yield strength, ultimate tensile strength, and uniform elongation of the high-Mn steel (base material) were approximately 230 HV, 494 MPa, 841 MPa, and 52%, respectively. From RHAZ to CGHAZ, the strength and hardness showed a decreasing trend. Compared with the CGHAZ, which had a uniform elongation of 59%, the RHAZ had a relatively lower uniform elongation of 51%. The yield strength in the RHAZ and THAZ was consistent with the change in recrystallization fraction, while in the CGHAZ, it satisfied the Hall–Petch relationship with grain size. The Hall–Petch coefficient was determined to be 488 MPa∙μm^1/2^. In addition, compared with the base material, the Charpy impact absorbed energy at −196 °C decreased by 22% in the RHAZ, but slightly increased in the CGHAZ. This indicates that the theory of fine grain strengthening and toughening is not entirely applicable to the HAZ of the investigated high-Mn steel.

## Data Availability

Data are contained within the article.
